# Adipose Tissue Circadian Dysregulation Beyond BMI: Implications for Cardiometabolic Risk and Cardiovascular Disease

**DOI:** 10.3390/life16020305

**Published:** 2026-02-10

**Authors:** Maria-Daniela Tanasescu, Andrei-Mihnea Rosu, Alexandru Minca, Maria-Mihaela Grigorie, Delia Timofte, Dorin Ionescu

**Affiliations:** 1 Department of Semiology-Emergency University Hospital, Carol Davila University of Medicine and Pharmacy, 022328 Bucharest, Romania; maria.tanasescu@umfcd.ro (M.-D.T.); dorin.ionescu@umfcd.ro (D.I.); 2 Department of Cardiology, Prof. Dr. Agrippa Ionescu Emergency Hospital, 077015 Balotesti, Romania; 3 Department of Dentistry, Discipline of Endodontics, Faculty of Dentistry, Carol Davila University of Medicine and Pharmacy, 020021 Bucharest, Romania; maria.grigorie@umfcd.ro; 4 Department of Dialysis, Bucharest Emergency University Hospital, 050098 Bucharest, Romania; delia.timofte@gmail.com

**Keywords:** circadian rhythm, adipose tissue, chronodisruption, visceral adiposity, insulin resistance, inflammation, cardiometabolic risk, cardiovascular disease

## Abstract

Cardiometabolic and cardiovascular risks are commonly assessed using body mass index (BMI) and static measures of adiposity; however, individuals with similar BMI frequently exhibit markedly different metabolic and cardiovascular outcomes. This heterogeneity reflects not only differences in fat distribution but also variation in adipose tissue function and its temporal regulation. Adipose tissue contains intrinsic circadian clocks that coordinate daily rhythms in lipid storage and mobilization, insulin sensitivity, adipokine secretion, and immune activity in alignment with sleep–wake and feeding–fasting cycles. Circadian misalignment, as occurs with shift work, irregular sleep, or mistimed food intake, disrupts this coordination and promotes adipose tissue dysfunction characterized by impaired rhythmic lipid handling, altered endocrine signaling, inflammation, fibrosis, and oxidative stress. Emerging evidence suggests that circadian dysregulation may differentially affect adipose depots, including visceral, epicardial, and perivascular fat, thereby linking chronodisruption to insulin resistance, endothelial dysfunction, atherosclerosis, heart failure phenotypes, and arrhythmia susceptibility. This narrative review synthesizes human, experimental, and translational studies examining adipose tissue circadian regulation as a functional determinant of cardiometabolic and cardiovascular risk beyond BMI. We also discuss the clinical implications of circadian-informed strategies, including chrononutrition and time-restricted eating, as potential tools to improve risk stratification and cardiometabolic health.

## 1. Introduction

Obesity is most commonly defined and classified using body mass index (BMI), an anthropometric measure derived from body weight and height that remains central to clinical practice and population-based research. While BMI is simple and widely applicable, substantial evidence indicates that it provides an incomplete assessment of adiposity-related health risk. Large population studies and meta-analyses have shown that BMI frequently misclassifies individuals with excess body fat but normal body weight, as well as individuals with elevated BMI who do not exhibit excess adiposity or metabolic impairment [1,2]. These limitations arise from BMI’s inability to distinguish between fat and lean mass and to capture heterogeneity in adipose tissue distribution, thereby restricting its value for cardiometabolic risk stratification.

Over the past two decades, adipose tissue has been redefined from a passive energy storage site to a biologically active organ with a fundamental role in cardiometabolic and cardiovascular health. Adipose tissue regulates lipid and glucose homeostasis and exerts endocrine and immune functions through the secretion of adipokines and inflammatory mediators. In obesity, adipose tissue undergoes maladaptive remodeling characterized by insulin resistance, chronic low-grade inflammation, altered adipokine secretion, and increased release of free fatty acids, processes that directly contribute to the development of type 2 diabetes and cardiovascular disease [3]. At the cellular level, adipocyte dysfunction—manifested by impaired lipid handling, mitochondrial stress, and sustained inflammatory signaling—has emerged as a key mechanistic link between excess adiposity and adverse cardiovascular outcomes [4]. In this context, cardiometabolic risk emerges as a function of adipose tissue quality as much as quantity.

An additional, and increasingly recognized, determinant of adipose tissue function is circadian regulation. In mammals, endogenous circadian clocks coordinate daily rhythms in physiology and behavior through a central pacemaker located in the suprachiasmatic nucleus and peripheral clocks expressed across metabolic tissues, including white and brown adipose tissue. Adipose tissue contains an intrinsic circadian clock that regulates lipid storage and mobilization, insulin sensitivity, thermogenesis, and adipokine secretion, thereby aligning metabolic processes with feeding–fasting cycles and daily energy demands [5,6]. Consistent with this role, a substantial proportion of adipose tissue gene expression follows circadian oscillations, thus accentuating the importance of temporal organization for normal adipose tissue metabolism [6].

Disruption of circadian rhythms has been consistently associated with adipose tissue dysfunction and metabolic disease. Behavioral and environmental factors such as shift work, irregular sleep–wake patterns, and mistimed food intake can desynchronize central and peripheral clocks, leading to circadian misalignment. Under these conditions, adipose tissue exhibits impaired insulin sensitivity, dysregulated lipid handling, altered adipokine secretion, and activation of inflammatory and oxidative stress pathways, contributing to systemic insulin resistance and increased cardiometabolic risk. It must be noted that these alterations may occur independently of changes in total adipose mass, suggesting that circadian dysregulation primarily affects adipose tissue quality rather than quantity [6,7].

Circadian misalignment has also been linked to oxidative stress and inflammatory signaling pathways that are central to the pathogenesis of metabolic and cardiovascular disease. Adipose tissue normally displays tightly regulated daily oscillations in lipid metabolism and endocrine activity. Disruption of these rhythms amplifies redox imbalance and inflammatory responses within adipose depots, further impairing adipocyte function and insulin signaling. Oxidative stress represents a shared pathophysiological mechanism underlying obesity, type 2 diabetes, and cardiovascular disease, and experimental and human studies indicate that circadian disruption intensifies these processes, thereby accelerating cardiometabolic complications [8].

Epidemiological and experimental data further support a link between circadian misalignment and adverse metabolic outcomes. Shift work and irregular eating patterns are associated with increased prevalence of obesity, insulin resistance, and type 2 diabetes. In humans, food intake at inappropriate circadian phases impairs glucose tolerance and reduces insulin sensitivity, reflecting a mismatch between behavioral rhythms and endogenous metabolic clocks. Adipose tissue appears to have a key mediating role in these effects, as circadian disruption promotes immune cell infiltration and cytokine production within adipose depots, a process often described as metabolic inflammation or “metaflammation” [9,10].

All in all, these findings indicate that adipose tissue dysfunction in cardiometabolic disease cannot be fully understood without consideration of circadian regulation. While BMI and other static measures provide information on body size and fat burden, they fail to capture the temporal organization of adipose tissue metabolism, endocrine signaling, and inflammatory activity. Circadian dysregulation, therefore, represents a critical and under-recognized dimension of adipose tissue biology with direct implications for cardiometabolic and cardiovascular disease development. This narrative review examines adipose tissue circadian dysregulation as a functional determinant of cardiometabolic and cardiovascular disease risk beyond BMI and static measures of adiposity.

## 2. Methods

We sought to evaluate the current evidence regarding the role of circadian regulation in adipose tissue biology and its contribution to cardiometabolic and cardiovascular risk beyond traditional anthropometric measures such as body mass index. Specifically, we examined how circadian misalignment affects adipose tissue function across distinct depots, the mechanistic links to metabolic and inflammatory pathways, and the translational relevance to cardiovascular disease phenotypes and potential chronotherapeutic interventions.

To address this aim, a comprehensive literature search was conducted in PubMed/MEDLINE, Scopus, and Web of Science from database inception through December 2025. The search strategy combined Medical Subject Headings (MeSH) and free-text keywords, including: (“circadian rhythm” OR “circadian clock” OR “clock genes” OR “chronodisruption” OR “circadian misalignment” OR “shift work”) AND (“adipose tissue” OR “adipocytes” OR “visceral adipose tissue” OR “subcutaneous adipose tissue” OR “epicardial adipose tissue” OR “perivascular adipose tissue” OR “brown adipose tissue”) AND (“insulin resistance” OR “metabolic dysfunction” OR “inflammation” OR “lipid metabolism” OR “cardiovascular disease” OR “atherosclerosis” OR “heart failure” OR “arrhythmia”).

Additional relevant studies were identified through manual screening of reference lists from key primary publications, recent systematic reviews, and meta-analyses.

Eligible sources included: (a) original research articles encompassing observational studies, randomized controlled trials, and mechanistic experimental studies; (b) systematic reviews and meta-analyses; (c) authoritative narrative reviews providing conceptual or mechanistic frameworks; and (d) relevant clinical and consensus guidelines. Articles published in English were prioritized. Case reports, editorials, and conference abstracts were excluded unless they provided unique mechanistic insights not captured in the peer-reviewed literature.

Given the narrative and integrative nature of this review, no formal risk-of-bias assessment or quantitative quality scoring was performed. Instead, study inclusion prioritized mechanistic insight, human and translational relevance, and conceptual contribution to understanding circadian regulation of adipose tissue function and cardiometabolic risk beyond static anthropometric measures.

## 3. Limitations of BMI and Static Adiposity Measures

### 3.1. BMI as a Static Descriptor of a Dynamic Tissue

BMI remains the most widely used criterion for defining obesity, yet it provides only a static, cross-sectional estimate of body size and fails to capture the marked biological heterogeneity observed among individuals with similar BMI values. In particular, BMI does not distinguish fat mass from lean mass or account for inter-individual differences in adipose tissue distribution, limitations that substantially weaken its ability to estimate cardiometabolic risk at the individual level. These shortcomings are reflected in the recognition of distinct obesity phenotypes, including metabolically healthy obesity (MHO) and metabolically unhealthy normal weight (MUNW), illustrating that BMI-based categories often fail to align with underlying metabolic status [11].

The concept of MHO emerged from observations that a subset of individuals with obesity lack overt cardiometabolic abnormalities at a given time point. Although definitions vary, MHO is generally characterized by the absence of major metabolic disturbances such as dyslipidemia, insulin resistance, hyperglycemia, and hypertension. However, the absence of consensus criteria has resulted in wide variability in reported prevalence and limited comparability across studies. As a result, MHO should not be interpreted as a benign or stable condition, as obesity is associated with adverse health outcomes that extend beyond conventional cardiometabolic risk factors [11,12].

A critical limitation of BMI-based classification is its inability to capture the dynamic nature of metabolic health over time. Longitudinal studies indicate that MHO frequently represents a transient phenotype rather than a stable state [13]. Evidence also shows that a substantial proportion of individuals initially classified as MHO develop metabolic abnormalities during follow-up, underscoring that cardiometabolic risk can evolve independently of BMI-defined obesity [14]. Similarly, a three-year follow-up study in a Mexican cohort reported that approximately one-third of participants transitioned between metabolic phenotypes, with the highest rate of change observed among those initially classified as MHO [15]. Thus, it is safe to assume that metabolic health exists along a continuum and can shift over clinically relevant timeframes—changes that static anthropometric measures such as BMI cannot anticipate.

The existence of MHO and MUNW, together with the frequent instability of metabolic phenotypes over time, shows BMI’s limitation as a static descriptor of a biologically and clinically dynamic condition. This temporal instability provides a compelling rationale for moving beyond BMI-only assessments when investigating mechanisms and modifiers of cardiometabolic risk, including the role of time-dependent regulatory processes such as circadian organization of adipose tissue function, discussed in subsequent sections.

### 3.2. Imaging-Based Adiposity: Improved Structure, Limited Functional Insight

Recognition of BMI’s limitations has prompted increasing use of imaging-based approaches to directly quantify adipose tissue distribution. Modalities such as computed tomography (CT), magnetic resonance imaging (MRI), and echocardiography allow precise assessment of visceral and ectopic fat depots, providing substantially greater anatomical resolution than anthropometric indices alone. Imaging-derived measures of visceral adipose tissue (VAT) and epicardial adipose tissue (EAT) have been shown to improve cardiometabolic risk stratification compared with BMI, particularly by identifying high-risk phenotypes among both obese and normal-weight individuals [11].

Among ectopic depots, EAT has received particular attention because of its close anatomical relationship with the myocardium and coronary vasculature. Translational and clinical studies indicate that EAT expansion is associated with local inflammation, myocardial fibrosis, and impaired diastolic function, especially in individuals with type 2 diabetes [16]. Imaging-based quantification of EAT therefore captures regionally relevant adiposity with direct pathophysiological implications for cardiovascular disease, beyond what can be inferred from generalized measures of obesity.

Prospective population data further support the clinical relevance of imaging-defined adiposity. In the Framingham Heart Study, directly measured VAT was independently associated with incident cardiovascular disease and cancer after adjustment for BMI and traditional risk factors, whereas subcutaneous adipose tissue was not [17].

Despite these advances, imaging-based metrics remain inherently structural. While they quantify adipose tissue volume or thickness with high precision, they provide limited insight into adipose tissue function, inflammatory activity, metabolic flexibility, or temporal organization [11,16,17]. Consequently, although imaging refines risk stratification beyond BMI, it remains insufficient to explain phenotype instability or disease progression over time. Thus, the need to integrate functional and temporal frameworks, particularly the circadian regulation of adipose tissue biology, appears.

## 4. Circadian Organization of Metabolic Homeostasis: A Focused Overview

### 4.1. Central and Peripheral Circadian Systems

Metabolic homeostasis is regulated by a hierarchical circadian system composed of a central pacemaker located in the suprachiasmatic nucleus (SCN) of the hypothalamus and a network of peripheral clocks distributed across metabolic tissues. This system provides temporal structure to physiology, allowing metabolic processes to anticipate daily cycles of activity, feeding, and rest, thereby supporting efficient energy utilization and cardiometabolic function. The SCN functions as the principal timekeeper by aligning internal rhythms with the external light–dark cycle. Photonic signals detected by intrinsically photosensitive retinal ganglion cells are transmitted directly to the SCN, constituting the dominant entrainment signal for central circadian timing [18,19]. Beyond serving as a general synchronizing cue, light exposure exerts time-of-day-dependent effects on circadian alignment with important metabolic implications. Environmental light is the primary zeitgeber responsible for daily resetting of the central pacemaker, and inappropriate timing or intensity of light exposure can destabilize this process, promoting circadian misalignment between central and peripheral clocks. Experimental and translational studies indicate that such misalignment alters the temporal coordination of glucose regulation, insulin sensitivity, and energy metabolism, independent of sleep duration or caloric intake [6]. In humans, exposure to light during the biological evening or night is increasingly recognized as a contributor to chronodisruption, with downstream associations with obesity and metabolic dysfunction, consistent with impaired circadian regulation of adipose and other metabolic tissues [5,6]. These observations are in accordance with the view that light signaling represents an upstream regulator of metabolic rhythmicity rather than a secondary behavioral confounder [5,6,9].

The metabolic consequences of light exposure depend not only on its presence but also on its circadian timing. Light exposure during the biological evening or night induces phase delays of central circadian signals, including suppression and temporal displacement of melatonin secretion, thereby shifting internal biological timing relative to behavioral cycles [20]. This phase delay coincides with a circadian window characterized by reduced glucose tolerance, diminished insulin sensitivity, and altered lipid handling, increasing susceptibility to metabolic dysfunction when nutrient intake occurs at inappropriate circadian phases. Human experimental studies demonstrate that evening or nocturnal light exposure perturbs glucose and lipid regulation in a manner consistent with circadian misalignment, even in the absence of major changes in sleep duration or total caloric intake, supporting a mechanistic role for light-driven circadian disruption rather than purely behavioral mediation [20,21].

Consistent with these timing-dependent effects, alterations in the timing and intensity of light exposure are increasingly recognized as contributors to metabolic dysregulation through their effects on circadian alignment. Exposure to light at inappropriate circadian phases, particularly during the biological evening or night, can perturb glucose and lipid regulation in a manner consistent with circadian misalignment. These effects appear to depend more strongly on the timing and amplitude of light exposure than on photoperiod per se, reinforcing the importance of circadian phase–specific signaling. Such metabolic associations are observed even in the absence of major changes in sleep duration, supporting a mechanistic role for circadian disruption rather than purely behavioral mediation [5,6]. Once synchronized, the SCN coordinates peripheral clocks through integrated neural, endocrine, and physiological pathways, including rhythmic autonomic nervous system output, oscillations in body temperature, and hormonal signals such as glucocorticoids and melatonin. Through these outputs, temporal information is conveyed to peripheral organs [18,19,22]. Because peripheral metabolic tissues are also highly responsive to non-photic cues (particularly feeding–fasting cycles), mistimed food intake occurring in the context of delayed light-entrained central rhythms can partially uncouple peripheral clocks from SCN control. This central–peripheral desynchrony provides a mechanistic framework linking light-driven circadian misalignment to tissue-specific metabolic dysfunction, particularly within adipose and hepatic systems [20].

**Figure 1:**
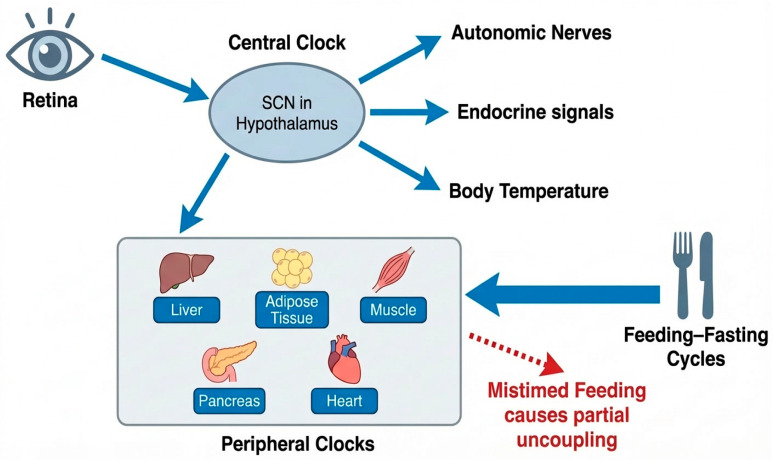
Central and peripheral circadian organization of metabolic homeostasis. The suprachiasmatic nucleus (SCN) in the hypothalamus functions as the central circadian pacemaker, receiving photic input from the retina and synchronizing peripheral clocks through autonomic, endocrine, and thermoregulatory pathways. Peripheral circadian clocks in metabolic tissues, including liver, adipose tissue, skeletal muscle, pancreas, and heart, are additionally entrained by feeding–fasting cycles. Mistimed feeding can partially uncouple peripheral clocks from central circadian control, contributing to metabolic dysregulation.

The hierarchical organization of central and peripheral circadian clocks, along with the major entrainment pathways relevant to metabolic regulation, is illustrated in Figure 1.

Peripheral circadian clocks are expressed in virtually all metabolic tissues, including liver, skeletal muscle, pancreas, cardiovascular tissues, and adipose depots, where they regulate local metabolic programs in a tissue-specific manner [22]. Although normally synchronized by the SCN, peripheral clocks retain sensitivity to non-photic zeitgebers, most notably feeding–fasting cycles and physical activity [18,19]. Under certain behavioral conditions, meal timing can selectively reset peripheral metabolic rhythms without fully realigning central circadian markers, resulting in partial dissociation between central and peripheral clocks [19].

Alignment between central and peripheral circadian systems is a key determinant of cardiometabolic health. Circadian misalignment, arising from shift work, irregular sleep timing, late chronotype (defined as a habitual preference for later circadian phase and sleep–wake timing), social jet lag, or mistimed food intake, has been consistently associated with increased risk of obesity, insulin resistance, dyslipidemia, hypertension, inflammation, and cardiovascular disease, independent of sleep duration and other lifestyle factors [18,19]. Chronotype represents a dynamic phenotype influenced by age, environmental light exposure, and seasonal factors, and should be distinguished from the intrinsic circadian period, which remains relatively stable across the lifespan. These associations support the view that circadian organization represents an independent dimension of metabolic regulation rather than a secondary consequence of altered behavior or adiposity.

Among non-photic cues, feeding–fasting cycles serve as particularly potent synchronizers of peripheral metabolic clocks. Experimental and translational studies demonstrate that the timing of food intake strongly shapes rhythmic metabolic function across organs, in some cases independently of total caloric intake or macronutrient composition. Time-restricted eating, which consolidates daily caloric intake within a consistent 8–12 h window, reinforces rhythmic nutrient sensing and promotes coordinated oscillations in glucose and lipid metabolism without requiring caloric restriction [23,24].

Human studies further illustrate the physiological relevance of circadian timing of food intake. Eating later relative to endogenous circadian phase, rather than clock time, is associated with increased body fat and higher BMI, independent of energy intake, diet composition, physical activity, or sleep duration [25]. Such observations suggest that misalignment between feeding behavior and internal circadian timing favors metabolic inefficiency and energy storage.

In addition to the SCN, other hypothalamic nuclei involved in energy balance, glucose regulation, and autonomic control display intrinsic rhythmicity and integrate signals from both central and peripheral clocks. Through coordinated neural and humoral outputs, this distributed circadian network regulates glucose production, insulin secretion, lipid mobilization, and thermogenesis across the daily cycle [24,26].

Disruption of this temporal architecture alters communication between tissues and regulatory pathways, creating a metabolic environment prone to dysfunction even in the absence of overt changes in body mass. Within this framework, adipose tissue represents a particularly sensitive target of circadian perturbation, providing a mechanistic link between chronodisruption and cardiometabolic disease.

### 4.2. Adipose Tissue as a Peripheral Circadian Organ

Adipose tissue functions as an autonomous peripheral circadian organ that integrates systemic timing cues with local endocrine, metabolic, and immune outputs. Experimental and translational studies show that both white and brown adipose depots exhibit intrinsic circadian rhythmicity, with coordinated oscillations that persist independently of the central clock while remaining highly responsive to feeding–fasting cycles and hormonal signals [10,27]. This dual autonomy and sensitivity position adipose tissue as a critical interface between circadian organization and whole-body metabolic homeostasis.

**Figure 2:**
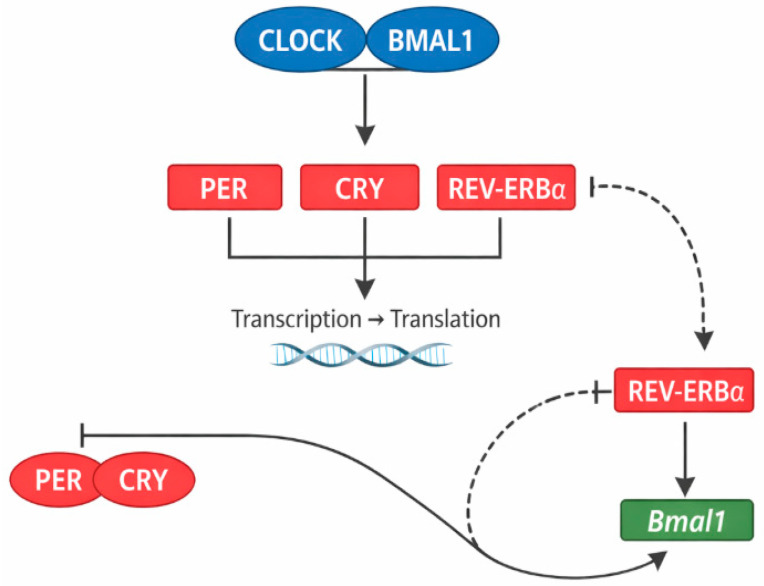
Molecular circadian clock mechanism in adipose tissue. The CLOCK–BMAL1 protein complex drives transcription of *Per*, *Cry*, and *Rev-erbα* genes. PER and CRY proteins inhibit CLOCK–BMAL1 activity in a negative feedback loop, while REV-ERBα suppresses *Bmal1* gene expression, forming a secondary regulatory loop.

This temporal coordination is driven by the core molecular circadian clock, composed of transcriptional feedback loops involving circadian locomotor output cycles kaput (CLOCK), brain and muscle ARNT-like 1 (BMAL1), period circadian protein (PER), cryptochrome (CRY), and nuclear receptor subfamily 1 group D member 1 (REV-ERBα) (Figure 2).

From an endocrine standpoint, adipose tissue displays marked daily variation in the secretion of adipokines and inflammatory mediators. Circulating levels of leptin, adiponectin, plasminogen activator inhibitor-1 (PAI-1), tumor necrosis factor-α, and interleukin-6 follow circadian patterns that mirror rhythmic activity within adipose depots [27]. These oscillations coincide with daily fluctuations in appetite regulation, insulin sensitivity, vascular tone, and thrombotic balance. When adipose circadian timing is disrupted, adipokine release becomes blunted or mistimed, favoring metabolic dysregulation and the development of low-grade inflammation.

At the metabolic level, adipose tissue clocks coordinate lipid storage and mobilization across the day–night cycle. Circadian regulation of lipolysis, fatty acid trafficking, and insulin responsiveness allows temporal partitioning between energy storage during feeding periods and substrate release during fasting states [24]. Disruption of this temporal organization, such as through irregular feeding schedules or shift work, impairs glucose tolerance, promotes ectopic lipid deposition, and exacerbates insulin resistance, even in the absence of significant changes in total caloric intake.

Adipose tissue appears particularly vulnerable to chronodisruption because of its strong dependence on behavioral and nutritional zeitgebers. Both experimental models and human studies indicate that mistimed feeding induces phase shifts and desynchronization of adipose circadian outputs relative to central and hepatic clocks [24,27]. In individuals with obesity, visceral adipose tissue shows altered temporal organization that correlates with components of the metabolic syndrome, supporting the notion that circadian misalignment amplifies adipose inflammation and cardiometabolic risk [28]. In parallel, immune cell populations within adipose depots exhibit time-dependent patterns of activity, suggesting that disruption of circadian control may directly contribute to inflammatory remodeling of adipose tissue [10].

Viewed in this context, adipose tissue operates as a circadian-regulated endocrine–metabolic–immune hub whose function depends not only on mass or location, but also on temporal integrity. Its heightened sensitivity to behavioral and environmental disruption provides a mechanistic link between circadian misalignment, insulin resistance, and chronic inflammation that extends beyond static measures of adiposity.

## 5. Depot-Specific Circadian Regulation of Adipose Tissue

### 5.1. Subcutaneous vs. Visceral Adipose Tissue

Human adipose tissue depots differ markedly in their circadian organization, with VAT showing greater vulnerability to rhythm disruption than subcutaneous adipose tissue (SAT), particularly in the setting of obesity. These depot-specific differences have important implications for insulin resistance, chronic inflammation, and cardiometabolic risk.

Direct 24 h profiling of human omental adipose tissue demonstrates that obesity profoundly alters the temporal expression of core clock genes, including CLOCK, BMAL1, PER1, CRY2, and REV-ERBα. Rather than simple attenuation, these changes reflect a reorganization of circadian rhythms within VAT. Altered rhythmicity of CRY2 and REV-ERBα correlates with waist circumference and other components of the metabolic syndrome, linking disruption of visceral circadian timing to clinically relevant metabolic risk [26].

Mechanistic human studies further indicate that circadian dysfunction in VAT is tightly coupled to inflammatory signaling. In individuals with obesity, omental adipose tissue exhibits impaired clock function characterized by reduced PER2 and REV-ERBα expression, loss of rhythmic BMAL1 chromatin binding, and lengthening of the circadian period. These alterations are driven, at least in part, by enhanced NF-κB (nuclear factor kappa B) activity, which directly interferes with clock gene regulation. Notably, pharmacological inhibition of NF-κB signaling restores circadian rhythmicity in human visceral adipose cells, supporting a causal role for inflammation in VAT clock disruption [29].

In contrast, SAT appears to retain greater circadian robustness. Comparative analyses of paired human SAT and VAT biopsies reveal higher PER1 expression and more stable temporal gene relationships in SAT, along with depot-specific associations between adiposity and clock gene expression [30]. These differences suggest that SAT is more resilient to metabolic stress, whereas VAT exhibits reduced circadian flexibility and heightened inflammatory sensitivity.

Additional human evidence reinforces this depot-specific pattern. Chronotype-associated circadian misalignment has been shown to translate into distinct inflammatory profiles in VAT but not SAT. In individuals with obesity, evening chronotypes exhibit significantly greater secretion of pro-inflammatory cytokines and chemokines from visceral fat, including interleukin-1 beta (IL-1β), interleukin-8 (IL-8), monocyte chemoattractant protein-1 (MCP-1), and macrophage inflammatory protein-1 beta (MIP-1β), despite similar anthropometric and metabolic characteristics across chronotypes [31]. These inflammatory changes are accompanied by altered expression of core clock genes in VAT, indicating that behavioral circadian misalignment preferentially amplifies visceral inflammatory risk.

Functional consequences of depot-specific circadian regulation are particularly evident at the level of insulin signaling. Ex vivo studies of human adipose tissue explants demonstrate a robust intrinsic circadian rhythm in insulin sensitivity in SAT, with peak responsiveness around midday and a marked decline during the night. In contrast, VAT shows no significant circadian rhythm in insulin signaling, reflecting a loss of temporal metabolic regulation within this depot. This absence of rhythmic insulin responsiveness in visceral fat provides a mechanistic link between VAT circadian dysfunction and systemic insulin resistance [32].

Circadian regulation of adipokine expression also differs between depots. Although adiponectin and its receptors display circadian oscillations in both SAT and VAT, visceral fat exhibits reduced amplitude and delayed phase relative to subcutaneous tissue. Increasing adiposity is associated with further attenuation of adiponectin rhythmicity, particularly in VAT, aligning depot-specific circadian disruption with features of the metabolic syndrome [33].

REV-ERBα (NR1D1) functions as a key circadian regulator of adipose metabolism, particularly in visceral depots where it represses lipogenic gene expression and promotes β-oxidation through interactions with BMAL1 and PPARγ (peroxisome proliferator-activated receptor gamma) [34]. NR1D1 deficiency leads to metabolic inflexibility, ectopic lipid accumulation, and impaired thermogenesis, with pronounced effects in VAT and brown fat. Human polymorphisms such as rs2314339 have been linked to obesity and insulin resistance, underscoring the gene’s clinical relevance [33]. Also, pharmacologic activation of REV-ERBα (e.g., SR9009) restores metabolic rhythmicity, suppresses inflammation, and improves insulin sensitivity, thus highlighting its potential as a therapeutic target in circadian disruption contexts such as shift work and obesity [34,35].

The principal depot-specific differences in circadian regulation, metabolic responsiveness, and inflammatory signaling between SAT and VAT, as derived from human studies, are summarized in Table 1.

### 5.2. Epicardial and Perivascular Adipose Tissue

EAT and perivascular adipose tissue (PVAT) are anatomically specialized fat depots with direct paracrine access to the myocardium and vascular wall, positioning them as local modulators of cardiovascular structure and function. Unlike subcutaneous depots, EAT lies in immediate contact with the myocardium and coronary arteries without an intervening fascial plane, permitting bidirectional exchange of adipokines, cytokines, and metabolic signals. PVAT, in turn, surrounds large and small vessels and exerts local effects on vascular tone and inflammation.

Human studies consistently demonstrate that EAT adopts a pro-inflammatory secretory profile in cardiovascular disease. In patients undergoing coronary artery bypass surgery, epicardial fat exhibits increased macrophage infiltration and higher expression of inflammatory adipocytokines compared with non-cardiac adipose depots, supporting its role as a local amplifier of myocardial and coronary inflammation [36]. Importantly, this phenotype remains modifiable: statin therapy has been associated with reduced interleukin-6 expression in epicardial fat, thus emphasizing the dynamic and clinically relevant nature of EAT signaling [36].

High-resolution transcriptomic analyses further refine this picture. Single-nucleus RNA sequencing of pericoronary EAT from patients with coronary artery disease identifies disease-specific adipocyte and macrophage subpopulations with altered immune and secretory programs. Dysregulation of circadian clock-related pathways within EAT adipocytes accompanies these changes, suggesting that temporal disorganization may contribute to sustained paracrine stress on adjacent coronary vessels [37].

The clinical consequences of EAT dysfunction extend beyond atherosclerosis. Integration of human imaging and pathological data supports the concept of an “epicardial adipose inflammatory triad,” in which EAT expansion and inflammation promote coronary atherosclerosis, atrial remodeling with arrhythmogenic vulnerability, and ventricular stiffening characteristic of heart failure with preserved ejection fraction [38]. Spatial heterogeneity across epicardial fat depots appears to influence the dominant phenotype, linking regional adipose inflammation to site-specific cardiac pathology.

Consistent with this framework, observational and mechanistic human studies implicate EAT as a contributor to atrial fibrillation. Pro-inflammatory and pro-fibrotic mediators released from epicardial fat infiltrate adjacent atrial myocardium, facilitating structural and electrical remodeling that lowers the threshold for arrhythmia initiation and maintenance [39]. These effects emphasize the relevance of EAT as a locally active adipose compartment rather than a passive bystander.

Building on the concept of depot-specific “local clocks,” PVAT has emerged as a time-structured paracrine organ capable of tuning vascular function across the day–night cycle. A key mechanistic advance is the identification of an intrinsic circadian clock within PVAT adipocytes, where BMAL1 transcriptionally regulates angiotensinogen expression and local angiotensin II availability. In brown-adipocyte–targeted Bmal1 or Agt knockout models, blood pressure is selectively reduced during the resting phase, producing a pronounced “super-dipper” phenotype; ex vivo, PVAT from wild-type mice increases aortic ring contractility in an endothelium-independent manner, an effect attenuated when Bmal1 is deleted in PVAT adipocytes [40].

Systemic clock disruption can further reprogram PVAT toward vascular dysfunction. In hepatocyte-specific Bmal1 knockout mice, liver clock perturbation alters circulating metabolic signals and remodels thoracic PVAT gene expression, including regulators of lipid handling and thermogenic programs. Under these conditions, PVAT selectively impairs endothelial function: acetylcholine-mediated vasorelaxation is reduced when PVAT remains attached, yet endothelial responsiveness is preserved once PVAT is removed [41]. This pattern supports a model in which mistimed systemic metabolism imprints on PVAT, reshaping the vasoactive environment presented to the vessel wall.

In atherometabolic disease, PVAT behaves as a double-edged layer. Under physiological conditions, it supports vascular homeostasis through anticontractile and anti-atherogenic mediators, as well as thermogenic lipid clearance. In obesity and metabolic dysfunction, PVAT becomes inflamed, loses thermogenic capacity, and releases pro-inflammatory adipokines and cytokines that promote endothelial dysfunction and plaque progression [42]. Framed in temporal terms, PVAT and by extension peri-coronary and peri-vascular fat, acts as a circadian effector tissue whose time-dependent paracrine outputs may influence atherosclerosis trajectories and downstream phenotypes such as arrhythmia susceptibility and heart failure remodeling.

EAT and PVAT represent anatomically specialized adipose depots with direct paracrine access to the myocardium and vascular wall, positioning them as local modulators of cardiovascular structure and function. Unlike subcutaneous depots, EAT lies in direct contact with the myocardium and coronary arteries without a separating fascia, enabling bidirectional exchange of cytokines, adipokines, and metabolic signals. The principal circadian and paracrine features distinguishing epicardial and perivascular adipose tissue are summarized in Table 2.

## 6. Circadian Dysregulation as a Driver of Adipose Tissue Dysfunction

Circadian dysregulation promotes adipose tissue dysfunction through multiple, interrelated mechanisms that affect lipid handling, endocrine signaling, and immune remodeling. In addition to environmental and behavioral drivers, interindividual genetic variation in circadian regulatory pathways modulates susceptibility to circadian dysregulation and its cardiometabolic consequences. Core clock genes, including CLOCK and BMAL1, constitute important components of the molecular circadian machinery and regulate metabolic homeostasis through transcriptional networks that integrate hypothalamic and peripheral signals. Genetic disruption or perturbation of these clock components alters glucose and lipid regulation, energy balance, and cardiometabolic risk, in some cases independently of overall adiposity [6,24,26]. Such genetic susceptibility is likely to interact with environmental circadian challenges, such as light exposure, shift work, or mistimed feeding, thereby contributing to heterogeneity in adipose tissue dysfunction and cardiometabolic outcomes under similar behavioral conditions.

### 6.1. Loss of Rhythmic Lipid Handling

Adipose tissue lipid storage and mobilization are intrinsically time-structured processes governed by circadian regulation. In humans, a defined subset of circulating and adipose-derived lipid species exhibits robust 24 h rhythmicity, reflecting coordinated temporal control of lipolysis, lipogenesis, and lipid trafficking. Lipidomic profiling of human subcutaneous adipose tissue and matched serum samples indicates that approximately 8% of lipid metabolites oscillate diurnally under metabolically healthy conditions. These rhythms are markedly disrupted in type 2 diabetes, with reduced amplitudes, phase shifts, and loss of synchrony between adipose tissue and circulating lipid pools, despite relatively stable mean lipid concentrations across the day. Time-resolved analyses reveal temporal disorganization affecting phospholipids, sphingolipids, and glycerolipid intermediates, indicating impaired coordination between adipose lipid handling and systemic lipid availability [43].

Circadian misalignment itself is sufficient to induce maladaptive lipid metabolic programs in humans. Experimental inversion of the behavioral cycle rapidly alters transcriptional pathways regulating fatty acid uptake and metabolism, accompanied by reduced insulin sensitivity and increased circulating free fatty acids [44]. Such behavioral misalignment often reflects underlying light-driven circadian phase delays, particularly those induced by evening or nocturnal light exposure that suppresses melatonin signaling, thereby desynchronizing central circadian timing cues from adipocyte lipid-metabolic clocks.

Mechanistic insight from experimental models further clarifies the consequences of disrupted adipose clocks. In mice with adipose-specific deletion of the core clock component BMAL1, diurnal variation in lipolysis is abolished, resulting in flattened free fatty acid and glycerol release profiles and increased triglyceride accumulation within adipocytes [45]. Loss of rhythmic lipid turnover is accompanied by elevated circulating lipid levels and ectopic fat deposition in the liver and skeletal muscle, indicating a failure of adipose tissue to buffer systemic lipid fluxes. In parallel, reduced adipogenic capacity and impaired adipose tissue expandability further redirect excess lipids toward non-adipose tissues under metabolic stress [46].

### 6.2. Adipokine Rhythmicity and Metabolic Signaling

Adipose tissue functions as a circadian-regulated endocrine organ, with adipokine secretion following defined daily rhythms that coordinate metabolic and inflammatory signaling. Circadian misalignment disrupts both the timing and amplitude of adipokine release, contributing to metabolic dysfunction independently of fat mass.

Leptin exhibits a well-characterized diurnal rhythm in humans, with higher nocturnal and lower daytime concentrations reflecting energy storage status. Circadian disruption and insufficient sleep attenuate this rhythmicity, producing flattened or phase-shifted leptin profiles. Even when mean leptin levels remain unchanged, loss of temporal signaling is associated with impaired satiety control and increased energy intake, consistent with a form of circadian leptin resistance. Adiponectin, an insulin-sensitizing and anti-inflammatory adipokine, also displays circadian variation under physiological conditions. Sleep disturbance and circadian misalignment are associated with reduced adiponectin levels, particularly in individuals with obesity, further compromising insulin sensitivity and favoring pro-inflammatory metabolic signaling [47,48].

Melatonin signaling represents a key upstream regulator linking circadian timing to adipokine rhythmicity and metabolic control. Melatonin secretion is tightly coupled to the light–dark cycle, peaks during the biological night, and exhibits an inverse temporal relationship with insulin secretion and glucose tolerance in humans. Disruption of melatonin rhythms by evening or nocturnal light exposure shifts internal circadian phase and alters downstream metabolic signaling pathways, including those governing leptin sensitivity and glucose handling [20].

Circadian disruption additionally reshapes the inflammatory secretory profile of adipose tissue. In human visceral fat, evening chronotype is associated with increased release of pro-inflammatory cytokines and chemokines, including IL-1β, IL-8, MCP-1, and MIP-1β, despite comparable anthropometric and metabolic characteristics [31]. These changes coincide with altered clock gene expression, linking local circadian disruption to inflammatory adipokine output.

Interindividual genetic variation in melatonin signaling further modulates susceptibility to these endocrine and metabolic effects. A common variant near the melatonin receptor gene MTNR1B (encoding the MT2 receptor) is robustly associated with elevated fasting plasma glucose, impaired early insulin secretion, and increased risk of type 2 diabetes across multiple large human cohorts, independent of obesity. MTNR1B is expressed in central circadian regulatory regions, including the suprachiasmatic nucleus, as well as in human pancreatic islets and beta cells, providing a mechanistic link between light-regulated melatonin signaling, circadian timing, and metabolic control. These findings support the concept that genetic variation in melatonin receptor signaling can influence individual vulnerability to metabolic consequences of circadian misalignment, including altered adipokine signaling and glucose homeostasis [49].

Human interventional studies indicate that temporal realignment can partially reverse these endocrine and inflammatory alterations; time-restricted eating (TRE) reduces circulating leptin and TNF-α (tumor necrosis factor alpha) levels, with more variable effects on IL-6 (interleukin-6) and adiponectin, despite modest weight loss [50]. Leptin appears particularly sensitive to temporal interventions, especially in individuals with overweight or obesity. In a randomized trial, early time-restricted eating produced greater reductions in leptin concentrations than late eating under comparable caloric restriction, highlighting sensitivity to circadian phase alignment rather than energy balance alone [51]. By contrast, circadian disruption through night-shift work produces opposing endocrine and inflammatory effects. In women exposed to night shifts, disturbed melatonin rhythms are accompanied by increased leptin concentrations and elevations in pro-inflammatory cytokines, including TNF-α, IL-1β, and IL-6, independent of long-term changes in body composition [52]. Therefore, it is safe to conclude that both environmental circadian disruption and genetic variation in melatonin signaling pathways shape adipokine rhythmicity and metabolic risk in humans [20,47].

These key features of adipokine circadian regulation and their disruption are summarized in Table 3.

### 6.3. Immune and Fibrotic Remodeling

Circadian regulation plays a central role in coordinating immune cell behavior within adipose tissue, and its disruption promotes a shift from adaptive immune surveillance toward chronic inflammatory remodeling. Experimental models of prolonged circadian misalignment demonstrate that disruption of light–dark cycles induces immune activation in both visceral and subcutaneous adipose depots, characterized by macrophage infiltration, crown-like structure formation, and loss of normal time-of-day-dependent gene regulation. These immune changes accompany adipocyte hypertrophy and precede overt metabolic dysfunction, indicating that immune remodeling represents an early response to circadian disruption [53].

Immune cell trafficking and function within adipose tissue are tightly regulated by circadian mechanisms. Regulatory T cells (Tregs) residing in visceral adipose tissue exhibit robust, cell-intrinsic circadian oscillations that are absent in lymphoid tissues, governing their activation state, metabolic fitness, and survival within the adipose microenvironment. Disruption of core clock components in Tregs abolishes diurnal variation in adipose tissue lipolysis and promotes constitutive immune activation, identifying circadian programming of tissue-resident immune cells as a key determinant of adipose immune homeostasis [54]. Sustained circadian misalignment shifts this balance toward chronic inflammation and fibrotic remodeling. In long-term models of shiftwork-like circadian disruption, adipose tissue shows upregulation of inflammatory and extracellular matrix-related gene programs, increased collagen deposition, and suppression of rhythmic transcriptional activity [53]. These fibrotic changes reduce tissue plasticity, constrain adipocyte expansion, and amplify inflammatory signaling, thereby accelerating adipose dysfunction.

Human data support the relevance of these pathways. Molecular profiling of abdominal subcutaneous adipose tissue following endurance exercise reveals rapid, intensity-dependent regulation of immune and extracellular matrix gene networks, including programs linked to angiogenesis, matrix remodeling, and reduced pro-inflammatory macrophage signaling [55]. Rather than being static, human adipose tissue remains responsive, with the capacity to reshape its immune and structural landscape when exposed to physiological stimuli.

Macrophages represent a critical interface linking circadian disruption to immune and fibrotic remodeling. These cells possess autonomous circadian clocks that regulate lipid and glucose metabolism in a time-of-day-dependent manner, thereby shaping inflammatory responsiveness within adipose tissue. Disruption of macrophage clock genes alters metabolic programming, favoring glycolytic, pro-inflammatory polarization with sustained cytokine production and impaired resolution of inflammation. Through secretion of matrix-modifying enzymes and profibrotic mediators, chronically activated macrophages promote extracellular matrix accumulation and loss of tissue flexibility [56].

Hypoxia further reinforces immune and fibrotic remodeling under circadian dysregulation. Altered oxygen availability disrupts core clock gene expression through hypoxia-inducible factors that interact directly with circadian transcriptional machinery, leading to dampened oscillatory amplitude and inter-tissue desynchrony. Hypoxia-driven circadian impairment is associated with enhanced inflammatory signaling, oxidative stress, and sympathetic activation, creating a feed-forward cycle that limits adipose tissue expandability and accelerates metabolic deterioration [57].

## 7. Circadian Misalignment and Obesity Phenotype Instability

### 7.1. From Metabolically Healthy to Unhealthy Obesity

MHO is increasingly recognized as a transient state rather than a stable phenotype. Although individuals with MHO may initially display preserved insulin sensitivity, limited visceral and ectopic fat accumulation, and maintained adipose tissue expandability, longitudinal studies show that a substantial proportion progress to metabolically unhealthy obesity (MUO) over time. This instability stresses the need to identify determinants of metabolic deterioration that extend beyond static measures of body mass.

A central feature of this transition is the gradual loss of adipose tissue adaptability. Under conditions of positive energy balance, effective expansion of subcutaneous adipose tissue limits lipid overflow to visceral depots and non-adipose tissues, thereby preserving insulin sensitivity. When this buffering capacity is exceeded or disrupted, lipid spillover ensues, accompanied by inflammation, adverse adipokine signaling, and metabolic impairment [11,12].

Circadian misalignment, driven by disrupted sleep–wake patterns, irregular feeding schedules, and attenuation of daily metabolic rhythms, appears to accelerate this process. Chronic exposure to late-night light, delayed sleep timing, and irregular feeding can progressively shift chronotype toward a later circadian phase, thereby destabilizing adipose tissue rhythmicity and weakening metabolic resilience. Sleep disruption and mistimed food intake impair the temporal regulation of adipose lipid handling and adipokine secretion, weakening coordination between energy storage and mobilization. Even in the absence of marked weight gain, loss of circadian control increases susceptibility to insulin resistance and low-grade inflammation [11]. As rhythmic adipose function deteriorates, subcutaneous fat becomes less effective as a metabolic buffer, favoring visceral fat expansion and ectopic lipid deposition—key features of MUO.

Clinical and mechanistic syntheses support this model of phenotype evolution. MHO is uncommon when stringent metabolic criteria are applied and frequently represents an intermediate stage preceding metabolic decline, characterized by worsening insulin sensitivity, increasing hepatic steatosis, and progressive adipose tissue dysfunction. Behavioral factors such as physical inactivity, weight gain, and impaired sleep contribute to this trajectory, highlighting the importance of temporal and lifestyle influences on metabolic resilience in obesity [58]. Cardiometabolic risk tracks more closely with fat distribution and adipose tissue dysfunction than with BMI itself, with visceral and ectopic fat strongly associated with progression toward dyslipidemia, glucose intolerance, and cardiovascular disease [59]. These observations are consistent with a framework in which disruption of adipose expandability and metabolic timing drives the shift from MHO to MUO.

Viewed through this lens, circadian misalignment functions as a permissive factor for phenotype conversion by eroding adipose tissue flexibility and metabolic resilience. The transition from metabolically healthy to unhealthy obesity reflects not simply cumulative fat gain, but a breakdown in the temporal organization of adipose tissue biology driven by disrupted sleep and feeding rhythms. This perspective reinforces the limitations of BMI-based categorization and supports circadian-aware approaches to preserving metabolic health in individuals with obesity.

### 7.2. Metabolically Unhealthy Normal Weight

MUNW describes individuals whose body mass index falls within the conventional normal range yet who exhibit insulin resistance, dyslipidemia, hypertension, hepatic steatosis, and elevated cardiometabolic risk. This phenotype exposes a fundamental limitation of BMI-based classification and reinforces the principle that metabolic health is shaped by adipose tissue function and fat distribution rather than body mass alone [11,12,59].

Genetic and metabolic data indicate that MUNW represents a biologically distinct phenotype rather than a statistical anomaly. Genome-wide analyses show that carriers of insulin resistance–associated alleles may have lower BMI but display increased visceral-to-subcutaneous fat ratios, reduced adiponectin levels, hepatic steatosis, and heightened risk of type 2 diabetes and coronary artery disease [60]. These features resemble aspects of partial lipodystrophy and point to impaired subcutaneous adipose tissue expandability as a central driver of metabolic dysfunction in the absence of overt obesity.

Circadian disruption appears to act as a critical, and often overlooked, modifier of this phenotype. Epidemiologic studies consistently link insufficient or irregular sleep, often driven by evening light exposure and delayed circadian phase, to impaired glucose tolerance, insulin resistance, hypertension, and dyslipidemia, even among individuals with normal body weight [61]. Such findings suggest that sleep loss and circadian misalignment can reveal latent metabolic vulnerability that remains hidden when risk assessment relies on BMI alone.

Mechanistic evidence supports a causal role for temporal misalignment. Experimental models of simulated shift work or behavioral circadian disruption induce rapid deterioration of cardiometabolic markers in healthy, lean adults, including impaired glucose regulation, inflammatory activation, endothelial dysfunction, and prothrombotic changes. These effects emerge within days and occur independently of changes in adiposity, underscoring the sensitivity of metabolic homeostasis to circadian timing [62].

In MUNW, chronodisruption likely contributes by destabilizing adipose tissue rhythmicity and endocrine signaling. Disruption of sleep–wake and feeding cycles in the context of delayed circadian phase impairs time-dependent regulation of lipolysis, adipokine secretion, and insulin sensitivity, reducing the capacity of subcutaneous adipose tissue to buffer energy excess. Lipid partitioning consequently shifts toward visceral and ectopic depots, promoting hepatic steatosis and systemic insulin resistance despite preserved BMI. Altered leptin signaling and reduced adiponectin further reinforce this trajectory [11,60].

The MUNW phenotype challenges the assumption that normal weight confers cardiometabolic protection. Individuals with adverse fat distribution and impaired circadian alignment may accumulate substantial risk while remaining undetected by conventional screening approaches. Framing MUNW as a circadian-sensitive metabolic phenotype highlights chronodisruption as a modifiable driver of disease and supports a shift toward functional, rather than anthropometric, definitions of metabolic risk.

## 8. Adipose Circadian Dysregulation and Cardiovascular Disease

Circadian regulation shapes cardiovascular physiology at systemic, vascular, and myocardial levels. Disruption of sleep–wake rhythms and chronic circadian misalignment are associated with increased risk of myocardial infarction and stroke, independent of traditional risk factors. Endogenous clocks within vascular cells, immune cells, and metabolic tissues coordinate daily oscillations in leukocyte trafficking, lipid handling, endothelial nitric oxide signaling, and thrombotic balance. When these rhythms are perturbed, endothelial activation, adhesion molecule expression, and monocyte recruitment to the arterial wall increase, accelerating atherogenesis [63].

At the lesion level, circadian control integrates immune and metabolic components of plaque biology. Leukocyte production and vascular recruitment follow diurnal patterns that are disrupted by sleep loss, shift work, and altered light exposure, sustaining inflammatory pressure on the vessel wall [64]. The heart itself contains an intrinsic circadian clock that coordinates myocardial metabolism, contractile performance, and responsiveness to neurohumoral cues. Core clock genes are rhythmically expressed in cardiomyocytes, and pressure overload selectively attenuates clock output without abolishing rhythmicity, reflecting reduced capacity to anticipate predictable daily fluctuations in workload and substrate availability [65]. Subsequent translational work has established circadian control as a determinant of myocardial metabolic flexibility, governing time-of-day-dependent shifts in glucose and lipid utilization, triglyceride handling, and NAD^+^ metabolism. Disruption of this temporal organization, through genetic clock perturbation or behavioral misalignment, impairs insulin, β-adrenergic, and mTOR signaling, increasing vulnerability to cardiac dysfunction and helping explain diurnal variation in heart failure symptoms and adverse events [66].

EAT forms a critical interface between circadian metabolism and heart failure pathophysiology, as it enables direct paracrine and metabolic interaction with cardiac tissue [62]. In health, it may buffer local energy demand and modulate coronary tone; in heart failure with preserved ejection fraction (HFpEF), EAT undergoes pathological expansion and inflammatory remodeling. Increased EAT volume associates with diastolic dysfunction, myocardial hypertrophy, elevated filling pressures, and adverse outcomes, independent of BMI [67]. Circadian dysregulation likely amplifies these effects by disrupting rhythmic adipose–myocardial signaling, impairing coordinated lipid flux and inflammatory resolution across the day. The convergence of myocardial clock dysfunction and maladaptive epicardial fat remodeling supports a chronometabolic view of HFpEF.

Cardiac electrical stability is also under circadian control. Myocardial repolarization exhibits endogenous rhythmicity governed by the cardiac molecular clock, with clock-controlled transcription regulating time-of-day-dependent expression of repolarizing ion channel components [68]. Disruption of this regulation abolishes QT interval rhythmicity and increases susceptibility to ventricular arrhythmias, identifying circadian misalignment as an arrhythmogenic substrate rather than a secondary phenomenon.

Autonomic regulation provides an additional link. Sympathetic and parasympathetic activity oscillate across the day, with circadian disruption blunting this balance and producing sustained sympathetic predominance, reduced heart rate variability, and impaired vagal recovery [69]. Melatonin contributes to autonomic stability by dampening sympathetic tone and oxidative stress. Suppression of melatonin, particularly due to nighttime light exposure and circadian misalignment, may increase arrhythmic vulnerability. Metabolic signals intersect with these pathways: insulin resistance, leptin signaling, and visceral adiposity promote sympathetic overdrive, influencing both electrophysiology and vascular tone [70]. Important to add, such autonomic activation can precede overt cardiovascular disease, particularly under sleep or metabolic stress.

Rather than acting in isolation, disrupted cardiac circadian timing, autonomic imbalance, and adipose-derived metabolic and inflammatory signaling converge to shape cardiovascular risk. When circadian adipose regulation is lost and sympathetic tone predominates, vascular, myocardial, and electrical stability are progressively compromised, positioning chronodisruption as a modifiable driver of cardiovascular disease.

To synthesize these links, Table 4 summarizes key circadian mechanisms, adipose contributions, and cardiovascular consequences across major disease domains.

## 9. Translational Implications and Therapeutic Perspectives

### 9.1. Chrononutrition and Time-Restricted Feeding

Chrononutrition strategies, particularly time-restricted feeding (TRF), have gained attention as clinically applicable interventions capable of restoring metabolic rhythmicity independently of substantial weight loss. Aligning food intake with earlier circadian phases improves metabolic regulation by reinforcing endogenous rhythms in substrate utilization, hormonal signaling, and energy expenditure [71]. Also, several weeks of early time-restricted feeding (Etrf) improved insulin sensitivity, lowered fasting insulin concentrations, and reduced markers of oxidative stress and inflammation, despite minimal weight loss. These benefits were accompanied by improved diurnal alignment of glucose regulation, suggesting restoration of temporal coordination between nutrient intake and metabolic processing rather than simple caloric restriction [72]. Longer-term interventions indicate that TRF may also influence adipose tissue distribution and cardiometabolic risk. TRF reduced visceral adipose tissue and improved features of the metabolic syndrome compared with control diets, even when energy intake was similar between groups [73].

Concerns regarding the feasibility and safety of prolonged daily fasting have been addressed by recent clinical data, the results being that early, late, or self-selected time-restricted eating (TRE) schedules did not adversely affect sleep duration, sleep quality, mood, or quality of life in adults with overweight or obesity [74]. This absence of negative effects on sleep and psychosocial outcomes supports the long-term acceptability of TRE in routine cardiometabolic care.

Emerging evidence also indicates that the metabolic impact of TRE depends on the timing of the eating window relative to endogenous circadian rhythms. In a three-month randomized intervention stratified by chronotype, it was determined that early TRE combined with energy restriction produced greater reductions in fat mass, fasting glucose, metabolic age, and diastolic blood pressure than late TRE or energy restriction alone, despite similar weight loss across groups [75]. It is safe to suggest, based on these results, that early alignment of food intake with circadian metabolic peaks enhances adipose lipid handling and cardiometabolic efficiency.

Recent syntheses of human TRE interventions further support consistent improvements in body composition, visceral adiposity, and cardiometabolic risk markers, while highlighting interindividual variability related to chronotype, baseline metabolic status, and adherence [76]. Rather than acting solely through energy reduction, time-restricted eating, most notably when implemented earlier in the day, appears to improve adipose tissue function by restoring temporal alignment of metabolic processes, offering a clinically relevant alternative to calorie-focused dietary strategies.

### 9.2. Pharmacological Modulation of Circadian Metabolism

Pharmacological treatment of metabolic disease increasingly intersects with circadian biology, both through drug mechanisms of action and through time-dependent variation in absorption, distribution, and efficacy. Glucagon-like peptide-1 receptor agonists (GLP-1RAs) illustrate this interplay, as they engage pathways under strong circadian control, including insulin secretion, appetite regulation, gastrointestinal motility, and adipose tissue metabolism. GLP-1–based therapies provide coordinated benefits across glycemic control, body weight, and cardiometabolic risk, while also influencing hormonal and autonomic systems with pronounced diurnal variation [77].

The importance of timing is particularly evident for orally administered GLP-1Ras, as systemic exposure to oral semaglutide is highly sensitive to fasting duration before and after dosing, with even modest deviations from recommended schedules markedly reducing bioavailability [78]. This finding reflects the circadian regulation of gastric emptying, intestinal motility, and splanchnic blood flow, and provides a practical explanation for interindividual variability in treatment response observed in routine care.

More broadly, principles of chronotherapy are gaining relevance across endocrine pharmacology. Circadian rhythms influence hormone secretion, receptor sensitivity, intracellular signaling, and drug metabolism, thereby shaping both efficacy and adverse-effect profiles. Aligning pharmacological interventions with endogenous biological timing has improved outcomes in several endocrine contexts and may hold similar promise in obesity and cardiometabolic disease [79].

Viewed in this context, pharmacological modulation of circadian metabolism should be considered a temporally adaptable strategy that integrates drug choice, formulation, and dosing time. Incorporating chronotherapeutic principles into metabolic treatment algorithms offers a rational approach to enhancing therapeutic consistency and clinical benefit by synchronizing pharmacology with circadian physiology.

### 9.3. Toward Circadian-Informed Risk Stratification

Current cardiometabolic risk stratification relies largely on static measurements that fail to capture temporal variation in physiology and behavior. Accumulating human evidence indicates that circadian misalignment represents an independent and clinically relevant contributor to cardiometabolic risk. Experimental circadian disruption in healthy adults rapidly impairs glucose tolerance, increases inflammatory activity, induces endothelial dysfunction, and promotes a prothrombotic state, even in the absence of changes in body weight or adiposity [60].

Advances in circadian biomarker development now permit objective assessment of internal biological time in clinical and research settings. Transcriptomic signatures derived from peripheral blood can estimate circadian phase with high accuracy from a limited number of samples, enabling quantification of circadian alignment independent of self-reported sleep or behavioral timing. Such tools identify individuals whose internal clocks are misaligned with external demands, a state associated with elevated cardiometabolic risk even among BMI-normal or otherwise healthy populations [80].

Complementary approaches using multi-tissue transcriptomic and metabolomic profiling also support the concept of a systemic circadian phenotype linked to metabolic disease risk. These methods provide a mechanistic bridge between molecular circadian disruption and downstream abnormalities in insulin sensitivity, lipid metabolism, and inflammatory signaling. When integrated with imaging-based assessment of adipose distribution or ectopic fat burden, circadian biomarkers may refine risk stratification by identifying individuals in whom temporal dysregulation amplifies structural and metabolic susceptibility [81,82].

From a translational standpoint, circadian-informed risk assessment offers several advantages. It enables earlier identification of risk states that precede overt disease, supports personalization of preventive strategies such as meal timing, physical activity, and pharmacotherapy, and complements existing clinical, imaging, and omics-based tools within a precision medicine framework. Framing cardiometabolic risk as a function of both magnitude and timing may improve detection of vulnerable individuals and guide temporally targeted interventions aimed at preserving metabolic resilience.

## 10. Future Directions and Knowledge Gaps

Although circadian regulation of adipose tissue is increasingly recognized as a determinant of cardiometabolic risk, key gaps remain between mechanistic insight and clinical translation. Human data are still limited, particularly with respect to time-resolved, depot-specific analyses of visceral, epicardial, and perivascular adipose tissue. Most available evidence derives from animal models or cross-sectional studies, so there is a need for longitudinal human investigations that capture how adipose circadian dysregulation evolves across different metabolic states.

Translation into clinical practice also remains challenging. While circadian misalignment is emerging as an independent risk factor, objective assessment of circadian timing is not yet incorporated into routine care. Advances in transcriptomic and metabolomic biomarkers offer promising tools to estimate internal biological time, but issues of validation, standardization, and feasibility outside research settings must be addressed before widespread implementation.

Uncertainty also persists regarding causality. Whether circadian dysregulation precedes adipose tissue dysfunction or primarily accelerates its progression under metabolic stress remains unclear. Resolving this question is particularly relevant for individuals with metabolically healthy obesity or metabolically unhealthy normal weight, in whom risk may accumulate before overt disease becomes apparent. Integrative longitudinal studies combining behavioral, molecular, and imaging data will be essential to define critical windows for intervention.

From a therapeutic perspective, greater emphasis should be placed on timing rather than solely on treatment intensity. Responses to chrononutrition, behavioral interventions, and pharmacotherapy likely vary according to chronotype, sex, age, and baseline metabolic status, yet these factors are rarely considered in trial design. Incorporating circadian phase and behavioral context into future studies may improve treatment effectiveness without introducing new therapies.

More broadly, progress will require a shift away from purely static models of cardiometabolic risk. Integrating circadian organization into assessments of adipose tissue function offers a more dynamic framework that captures metabolic resilience and vulnerability over time. Bridging chronobiology with precision medicine has the potential to identify at-risk individuals earlier and guide interventions aimed at preserving metabolic health before irreversible damage occurs.

## 11. Conclusions

Circadian regulation of adipose tissue represents a critical dimension of cardiometabolic health that is not captured by static measures such as body mass index. Beyond its role in energy storage, adipose tissue operates as a temporally organized endocrine and immune organ, and disruption of this organization alters lipid handling, inflammatory tone, and metabolic signaling in ways that substantially influence disease risk.

Evidence reviewed here shows that circadian misalignment contributes to adipose tissue dysfunction across a range of phenotypes, from metabolically healthy obesity to cardiometabolically unhealthy normal weight. Depot-specific vulnerability, particularly within visceral, epicardial, and perivascular fat, provides a mechanistic link between chronodisruption and insulin resistance, cardiovascular remodeling, and arrhythmic risk, helping to explain why cardiometabolic outcomes often diverge from BMI-defined categories.

Important to note: circadian dysregulation is not a fixed trait. Interventions that restore temporal alignment, including chrononutrition, sleep optimization, and timing-aware pharmacotherapy, can improve metabolic regulation and adipose tissue function even in the absence of substantial weight loss. Emerging circadian biomarkers further raise the possibility of identifying individuals at risk earlier and tailoring prevention strategies to internal biological timing.

This review argues for a shift toward a functional, time-aware view of adipose biology in cardiometabolic research and clinical practice. Recognizing when metabolic processes are disrupted—rather than focusing exclusively on how much adipose tissue is present—may be essential for improving risk stratification, explaining phenotype instability, and guiding more effective, personalized interventions aimed at preserving metabolic and cardiovascular health.

## Figures and Tables

**Table 1:** Depot-specific circadian characteristics of subcutaneous and visceral adipose tissue in humans.

Domain	Subcutaneous Adipose Tissue	Visceral Adipose Tissue
Anatomical location	Located beneath the skin; peripheral adipose depot	Located within the abdominal cavity; surrounds the visceral organs
Baseline circadian organization	Relatively preserved circadian regulation across molecular and functional outputs	More prone to circadian disruption, particularly in obesity and inflammatory states
Response to metabolic stress	Greater resilience of circadian function with increasing adiposity	Early and pronounced circadian dysregulation in response to metabolic stress
Insulin sensitivity (time-of-day dependence)	Clear circadian rhythm, with higher sensitivity during daytime hours	Lacks consistent circadian rhythmicity, indicating impaired temporal metabolic control
Inflammatory profile	Lower basal inflammatory activity with limited interference in circadian regulation	Higher inflammatory activity that directly disrupts circadian regulatory pathways
Adipokine rhythmicity	Adipokine expression and signaling maintain circadian oscillations	Adipokine rhythms are frequently dampened or phase-shifted, particularly in obesity
Sensitivity to circadian misalignment	Less clearly affected in available human data	Highly sensitive, with amplified inflammatory and metabolic responses
Overall metabolic implication	Generally associated with less harmful metabolic profiles when circadian regulation is preserved	Strongly linked to insulin resistance, inflammation, and cardiometabolic risk when circadian control is impaired

**Table 2:** Key circadian and paracrine characteristics of epicardial and perivascular adipose tissue.

Domain	Epicardial Adipose Tissue	Perivascular Adipose Tissue
Anatomical proximity	Directly adjacent to the myocardium and coronary arteries without fascial separation	Surrounds blood vessels in close contact with the vascular wall
Circadian organization	Evidence of altered circadian structure accompanying inflammatory and metabolic remodeling in cardiovascular disease	Possesses an intrinsic peripheral clock that modulates vascular function in a time-of-day-dependent manner
Dominant paracrine effects	Releases inflammatory and pro-fibrotic mediators affecting myocardium and coronary biology	Releases vasoactive and inflammatory mediators influencing vascular tone and endothelial function
Inflammatory phenotype in disease	Pro-inflammatory profile in coronary artery disease, atrial fibrillation, and heart failure	Shift from protective to pro-inflammatory, pro-atherogenic phenotype in obesity
Major cardiovascular associations	Coronary atherosclerosis, atrial arrhythmias, and heart failure phenotypes	Atherosclerosis, blood pressure dysregulation, vascular stiffness
Conceptual role	Locally active depot amplifying myocardial vulnerability under metabolic and inflammatory stress	Circadian effector depot translating timing cues into vascular responses

**Table 3:** Circadian characteristics and disruption responses of selected adipokines.

Adipokine	Circadian Pattern	Effect of Circadian Disruption	Modulation by Temporal Intervention
Leptin	Peaks at night; lower during day; reflects energy storage status	Flattened or phase-shifted rhythm; impaired satiety signaling; increased appetite	Decreased by time-restricted eating (TRE), especially with early-phase alignment
Adiponectin	Displays daily oscillations; supports insulin sensitivity and anti-inflammatory signaling	Decreased levels, especially in obesity; impaired insulin sensitivity	Variable response to TRE; possible modest increase with improved circadian alignment
TNF-α	Exhibits rhythmic secretion; involved in inflammatory tone	Increased with circadian misalignment and night-shift work	Reduced by TRE interventions
IL-6	Rhythmic under physiological conditions	Elevated in circadian disruption (e.g., night-shift work)	TRE produces variable effects; some reduction is observed
IL-1β, IL-8, MCP-1, MIP-1β	Not fully characterized; secretion influenced by circadian phase	Elevated in visceral fat of evening chronotypes; associated with altered clock gene expression	Limited data; likely responsive to circadian realignment strategies

Abbreviations: TRE, Time-Restricted Eating; TNF-α, Tumor Necrosis Factor-alpha; IL-6, Interleukin-6; IL-1β, Interleukin-1 beta; IL-8, Interleukin-8; MCP-1, Monocyte Chemoattractant Protein-1; MIP-1β, Macrophage Inflammatory Protein-1 beta.

**Table 4:** Circadian Adipose Dysregulation and Cardiovascular Disease.

Disease Domain	Circadian Mechanisms	Adipose Contribution	Key Consequences	Clinical Phenotype
Atherosclerosis/CAD	Disrupted immune trafficking; impaired endothelial NO; prothrombotic shift	Visceral/PVAT inflammation; impaired lipid buffering	Endothelial dysfunction; plaque progression	CAD risk; diurnal clustering of events
Myocardial metabolism/HF	Attenuated cardiomyocyte clock output; loss of rhythmic substrate use	EAT expansion; local inflammation and hypoxia	Fibrosis; energetic inefficiency; diastolic dysfunction	HFpEF; exercise intolerance
EAT–myocardial crosstalk	Desynchrony between myocardial and adipose clocks	Pro-inflammatory adipokine signaling	Direct myocardial metabolic stress	Worsening diastolic function
Electrophysiology/arrhythmias	Loss of QT rhythmicity; altered ion channel expression	Adipokine-driven autonomic modulation	Electrical instability	Ventricular arrhythmias; sudden death
Autonomic regulation	Blunted sympathetic–parasympathetic oscillation	Leptin/insulin-mediated adrenergic drive	Reduced HRV; endothelial dysfunction	Hypertension; cardiometabolic progression

Abbreviations: CAD, coronary artery disease; EAT, epicardial adipose tissue; HF, heart failure; HFpEF, heart failure with preserved ejection fraction; HRV, heart rate variability; NO, nitric oxide; PVAT, perivascular adipose tissue; QT, QT interval.

## Data Availability

No new data were created or analyzed in this study. Data sharing is not applicable to this article.

## Abbreviations

The following abbreviations are used in this manuscript:

BMI	Body Mass Index
CAD	Coronary artery disease
CLOCK	Circadian locomotor output cycles kaput
CRY2	Cryptochrome circadian regulator 2
EAT	Epicardial adipose tissue
eTRF	Early time-restricted feeding
HF	Heart failure
HFpEF	Heart failure with preserved ejection fraction
MHO	Metabolically Healthy Obesity
MUNW	Metabolically Unhealthy Normal Weight
PVAT	Perivascular adipose tissue
SAT	Subcutaneous Adipose Tissue
SCN	Suprachiasmatic nucleus
TRE	Time-restricted eating
TRF	Time-restricted feeding
VAT	Visceral adipose tissue

## References

[1] Romero-Corral A., Somers V.K., Sierra-Johnson J., Thomas R.J., Collazo-Clavell M.L., Korinek J., Allison T.G., Batsis J.A., Sert-Kuniyoshi F.H., Lopez-Jimenez F. (2008). Accuracy of body mass index in diagnosing obesity in the adult general population. Int. J. Obes..

[2] Okorodudu D.O., Jumean M.F., Montori V.M., Romero-Corral A., Somers V.K., Erwin P.J., Lopez-Jimenez F. (2010). Diagnostic performance of body mass index to identify obesity as defined by body adiposity: A systematic review and meta-analysis. Int. J. Obes..

[3] Chait A., den Hartigh L.J. (2020). Adipose tissue distribution, inflammation and its metabolic consequences, including diabetes and cardiovascular disease. Front. Cardiovasc. Med..

[4] Giroud M., Jodeleit H., Prentice K.J., Bartelt A. (2021). Adipocyte function and the development of cardiometabolic disease. J. Physiol..

[5] Froy O., Garaulet M. (2018). The circadian clock in white and brown adipose tissue: Mechanistic, endocrine, and clinical aspects. Endocr. Rev..

[6] Stenvers D.J., Scheer F.A.J.L., Schrauwen P., la Fleur S.E., Kalsbeek A. (2019). Circadian clocks and insulin resistance. Nat. Rev. Endocrinol..

[7] Pagano E.S., Spinedi E., Gagliardino J.J. (2017). White adipose tissue and circadian rhythm dysfunctions in obesity: Pathogenesis and available therapies. Neuroendocrinology.

[8] Man A.W.C., Xia N., Li H. (2020). Circadian Rhythm in Adipose Tissue: Novel Antioxidant Target for Metabolic and Cardiovascular Diseases. Antioxidants.

[9] Oosterman J.E., Wopereis S., Kalsbeek A. (2020). The circadian clock, shift work, and tissue-specific insulin resistance. Endocrinology.

[10] Kolbe I., Oster H. (2019). Chronodisruption, metabolic homeostasis, and the regulation of inflammation in adipose tissues. Yale J. Biol. Med..

[11] Tanasescu M.-D., Rosu A.-M., Minca A., Rosu A.-L., Grigorie M.-M., Timofte D., Ionescu D. (2025). Beyond BMI: Rethinking Obesity Metrics and Cardiovascular Risk in the Era of Precision Medicine. Diagnostics.

[12] Blüher M. (2020). Metabolically Healthy Obesity. Endocr. Rev..

[13] Phillips C.M. (2017). Metabolically healthy obesity across the life course: Epidemiology, determinants, and implications. Ann. N. Y. Acad. Sci..

[14] Cho Y.K., Jung C.H. (2022). Metabolically healthy obesity: It is time to consider its dynamic changes. Cardiovasc. Prev. Pharmacother..

[15] Elías-López D., Vargas-Vázquez A., Mehta R., Cruz Bautista I., Del Razo Olvera F., Gómez-Velasco D., Almeda Valdes P., Aguilar-Salinas C.A., Metabolic Syndrome Study Group (2021). Natural course of metabolically healthy phenotype and risk of developing cardiometabolic diseases: A three years follow-up study. BMC Endocr. Disord..

[16] Gil-Millan P., Rives J., Sánchez-Quesada J.L., Pérez A. (2025). Epicardial Fat and Heart Failure in Type 2 Diabetes: Metabolism, Imaging and Novel Biomarkers—A Translational Perspective. J. Clin. Med..

[17] Britton K.A., Massaro J.M., Murabito J.M., Kreger B.E., Hoffmann U., Fox C.S. (2013). Body Fat Distribution, Incident Cardiovascular Disease, Cancer, and All-Cause Mortality. J. Am. Coll. Cardiol..

[18] Ansu Baidoo V., Knutson K.L. (2023). Associations between circadian disruption and cardiometabolic disease risk: A review. Obesity.

[19] Knutson K.L., Dixon D.D., Grandner M.A., Jackson C.L., Kline C.E., Maher L., Makarem N., Martino T.A., St-Onge M.-P., Johnson D.A. (2025). Role of circadian health in cardiometabolic health and disease risk: A scientific statement from the American Heart Association. Circulation.

[20] Dashti H.S., Jansen E.C., Zuraikat F.M., Dixit S., Brown M., Laposky A., Broussard J.L., Butler M.P., Creasy S.A., Crispim C.A. (2025). Advancing Chrononutrition for Cardiometabolic Health: A 2023 National Heart, Lung, and Blood Institute Workshop Report. J. Am. Heart Assoc..

[21] Centofanti S., Heilbronn L.K., Wittert G., Dorrian J., Coates A.M., Kennaway D., Gupta C., Stepien J.M., Catcheside P., Yates C. (2025). Fasting as an intervention to alter the impact of simulated night-shift work on glucose metabolism in healthy adults: A cluster randomised controlled trial. Diabetologia.

[22] Dibner C., Schibler U., Albrecht U. (2010). The mammalian circadian timing system: Organization and coordination of central and peripheral clocks. Annu. Rev. Physiol..

[23] Chaix A., Manoogian E.N.C., Melkani G.C., Panda S. (2019). Time-restricted eating to prevent and manage chronic metabolic diseases. Annu. Rev. Nutr..

[24] Kalsbeek A., la Fleur S., Fliers E. (2014). Circadian control of glucose metabolism. Mol. Metab..

[25] McHill A.W., Phillips A.J.K., Czeisler C.A., Keating L., Yee K., Barger L.K., Garaulet M., Scheer F.A.J.L., Klerman E.B. (2017). Later circadian timing of food intake is associated with increased body fat. Am. J. Clin. Nutr..

[26] Cedernaes J., Waldeck N., Bass J. (2019). Neurogenetic basis for circadian regulation of metabolism by the hypothalamus. Genes Dev..

[27] Zvonic S., Ptitsyn A.A., Conrad S.A., Scott L.K., Floyd Z.E., Kilroy G., Wu X., Goh B.C., Mynatt R.L., Gimble J.M. (2006). Characterization of peripheral circadian clocks in adipose tissues. Diabetes.

[28] Vieira E., Ruano E.G., Figueroa A.L.C., Aranda G., Momblan D., Carmona F., Gomis R., Vidal J., Hanzu F.A. (2014). Altered circadian organization of visceral adipose tissue is associated with metabolic syndrome. PLoS ONE.

[29] Maury E., Navez B., Brichard S.M. (2021). Circadian clock dysfunction in human omental fat links obesity to metabolic inflammation. Nat. Commun..

[30] Zanquetta M.M., Correa-Giannella M.L., Giannella-Neto D., Alonso P.A., Guimarães L.M., Meyer A., Villares S.M. (2012). Expression of clock genes in human subcutaneous and visceral adipose tissues. Chronobiol. Int..

[31] Zinna L., Verde L., Di Tolla M.F., Barrea L., Parascandolo A., D’Alterio F., Colao A., Formisano P., D’Esposito V., Muscogiuri G. (2025). Chronodisruption enhances inflammatory cytokine release from visceral adipose tissue in obesity. J. Transl. Med..

[32] Carrasco-Benso M.P., Rivero-Gutierrez B., Lopez-Minguez J., Anzola A., Diez-Noguera A., Madrid J.A., Lujan J.A., Martínez-Augustin O., Scheer F.A.J.L., Garaulet M. (2016). Human adipose tissue expresses intrinsic circadian rhythm in insulin sensitivity. FASEB J..

[33] Gómez-Abellán P., Gómez-Santos C., Madrid J.A., Milagro F.I., Campion J., Martínez J.A., Ordovás J.M., Garaulet M. (2010). Circadian expression of adiponectin and its receptors in human adipose tissue. Endocrinology.

[34] Zhang-Sun Z.-Y., Xu X.-Z., Escames G., Lei W.-R., Zhao L., Zhou Y.-Z., Tian Y., Ren Y.-N., Acuña-Castroviejo D., Yang Y. (2023). Targeting NR1D1 in organ injury: Challenges and prospects. Mil. Med. Res..

[35] Ribas-Latre A., Eckel-Mahan K. (2022). Nutrients and the Circadian Clock: A Partnership Controlling Adipose Tissue Function and Health. Nutrients.

[36] Baker A.R., da Silva N.F., Quinn D.W., Harte A.L., Pagano D., Bonser R.S., Kumar S., McTernan P.G. (2006). Human epicardial adipose tissue expresses a pathogenic profile of adipocytokines in patients with cardiovascular disease. Cardiovasc. Diabetol..

[37] Liu X., Yuan M., Zhao D., Zeng Q., Li W., Li T., Li Q., Zhuo Y., Luo M., Chen P. (2024). Single-nucleus transcriptomic atlas of human pericoronary epicardial adipose tissue in normal and pathological conditions. Arterioscler. Thromb. Vasc. Biol..

[38] Packer M. (2018). The epicardial adipose inflammatory triad: Coronary atherosclerosis, atrial fibrillation, and heart failure with preserved ejection fraction. Eur. J. Heart Fail..

[39] Conte M., Petraglia L., Cabaro S., Valerio V., Poggio P., Pilato E., Attena E., Russo V., Ferro A., Formisano P. (2022). Epicardial adipose tissue and cardiac arrhythmias: Focus on atrial fibrillation. Front. Cardiovasc. Med..

[40] Chang L., Xiong W., Zhao X., Fan Y., Guo Y., Garcia-Barrio M., Zhang J., Jiang Z., Lin J.D., Chen Y.E. (2018). Bmal1 in perivascular adipose tissue regulates resting phase blood pressure through transcriptional regulation of angiotensinogen. Circulation.

[41] Pati P., Valcin J.A., Zhang D., Neder T.H., Millender-Swain T., Allan J.M., Sedaka R., Jin C., Becker B.K., Pollock D.M. (2021). Liver circadian clock disruption alters perivascular adipose tissue gene expression and aortic function in mice. Am. J. Physiol. Regul. Integr. Comp. Physiol..

[42] Qi X.-Y., Qu S.-L., Xiong W.-H., Rom O., Chang L., Jiang Z.-S. (2018). Perivascular adipose tissue (PVAT) in atherosclerosis: A double-edged sword. Cardiovasc. Diabetol..

[43] Sinturel F., Chera S., Brulhart-Meynet M.-C., Paz Montoya J., Stenvers D.J., Bisschop P.H., Kalsbeek A., Guessous I., Jornayvaz F.R., Philippe J. (2023). Circadian organization of lipid landscape is perturbed in type 2 diabetic patients. Cell Rep. Med..

[44] Wefers J., van Moorsel D., Hansen J., Connell N.J., Havekes B., Hoeks J., van Marken Lichtenbelt W.D., Duez H., Phielix E., Kalsbeek A. (2018). Circadian misalignment induces fatty acid metabolism gene profiles and compromises insulin sensitivity in human skeletal muscle. Proc. Natl. Acad. Sci. USA.

[45] Shostak A., Meyer-Kovac J., Oster H. (2013). Circadian regulation of lipid mobilization in white adipose tissues. Diabetes.

[46] Shimba S., Ogawa T., Hitosugi S., Ichihashi Y., Nakadaira Y., Kobayashi M., Tezuka M., Kosuge Y., Ishige K., Ito Y. (2011). Deficient of a clock gene, brain and muscle Arnt-like protein-1 (BMAL1), induces dyslipidemia and ectopic fat formation. PLoS ONE.

[47] Chaput J.-P., McHill A.W., Cox R.C., Broussard J.L., Dutil C., da Costa B.G.G., Sampasa-Kanyinga H., Wright K.P. (2023). The Role of Insufficient Sleep and Circadian Misalignment in Obesity. Nat. Rev. Endocrinol..

[48] Wei Z., Chen Y., Upender R.P. (2022). Sleep Disturbance and Metabolic Dysfunction: The Roles of Adipokines. Int. J. Mol. Sci..

[49] Bouatia-Naji N., Bonnefond A., Cavalcanti-Proença C., Sparsø T., Holmkvist J., Marchand M., Delplanque J., Lobbens S., Rocheleau G., Durand E. (2009). A variant near MTNR1B is associated with increased fasting plasma glucose levels and type 2 diabetes risk. Nat. Genet..

[50] Turner L., Charrouf R., Martínez-Vizcaíno V., Hutchison A., Heilbronn L.K., Fernández-Rodríguez R. (2024). The Effects of Time-Restricted Eating versus Habitual Diet on Inflammatory Cytokines and Adipokines in the General Adult Population: A Systematic Review with Meta-Analysis. Am. J. Clin. Nutr..

[51] Habe B., Črešnovar T., Petelin A., Kenig S., Mohorko N., Jenko Pražnikar Z. (2025). Comparing the Influence of Early and Late Time-Restricted Eating with Energy Restriction on Cardiometabolic Markers, Metabolic Hormones and Appetite in Adults with Overweight/Obesity. Nutr. Metab..

[52] Cakan P., Yildiz S. (2020). Effects of Half- or Whole-Night Shifts on Physiological and Cognitive Parameters in Women. Am. J. Med. Sci..

[53] Xiong X., Lin Y., Lee J., Paul A., Yechoor V., Figueiro M., Ma K. (2021). Chronic Circadian Shift Leads to Adipose Tissue Inflammation and Fibrosis. Mol. Cell. Endocrinol..

[54] Xiao T., Langston P.K., Muñoz-Rojas A.R., Jayewickreme T., Lazar M.A., Benoist C., Mathis D. (2022). Tregs in Visceral Adipose Tissue Up-Regulate Circadian-Clock Expression to Promote Fitness and Enforce a Diurnal Rhythm of Lipolysis. Sci. Immunol..

[55] Ahn C., Zhang T., Rode T., Yang G., Chugh O.K., Ellis S., Ghayur S., Mehta S., Salzman R., Jiang H. (2025). Molecular Responses in Abdominal Subcutaneous Adipose Tissue after a Session of Endurance Exercise: Effects of Exercise Intensity. J. Physiol..

[56] Dar M.I., Hussain Y., Pan X. (2025). Roles of Circadian Clocks in Macrophage Metabolism: Implications in Inflammation and Metabolism of Lipids, Glucose, and Amino Acids. Am. J. Physiol. Endocrinol. Metab..

[57] Castillejos-López M., Romero Y., Varela-Ordoñez A., Flores-Soto E., Romero-Martínez B.S., Velázquez-Cruz R., Vázquez-Pérez J.A., Ruiz V., Gómez-Verján J.C., Rivero-Segura N.A. (2023). Hypoxia Induces Alterations in the Circadian Rhythm in Patients with Chronic Respiratory Diseases. Cells.

[58] Smith G.I., Mittendorfer B., Klein S. (2019). Metabolically Healthy Obesity: Facts and Fantasies. J. Clin. Investig..

[59] Neeland I.J., Poirier P., Després J.-P. (2018). Cardiovascular and Metabolic Heterogeneity of Obesity: Clinical Challenges and Implications for Management. Circulation.

[60] Yaghootkar H., Scott R.A., White C.C., Zhang W., Speliotes E., Munroe P.B., Ehret G.B., Bis J.C., Fox C.S., Walker M. (2014). Genetic Evidence for a Normal-Weight “Metabolically Obese” Phenotype Linking Insulin Resistance, Hypertension, Coronary Artery Disease, and Type 2 Diabetes. Diabetes.

[61] Knutson K.L. (2010). Sleep Duration and Cardiometabolic Risk: A Review of the Epidemiologic Evidence. Best Pract. Res. Clin. Endocrinol. Metab..

[62] McHill A.W., Melanson E.L., Wright K.P., Depner C.M. (2024). Circadian Misalignment Disrupts Biomarkers of Cardiovascular Disease Risk and Promotes a Hypercoagulable State. Eur. J. Neurosci..

[63] Maury E., Ramsey K.M., Bass J. (2010). Circadian Rhythms and Metabolic Syndrome: From Experimental Genetics to Human Disease. Circ. Res..

[64] McAlpine C.S., Swirski F.K. (2016). Circadian Influence on Metabolism and Inflammation in Atherosclerosis. Circ. Res..

[65] Young M.E., Razeghi P., Taegtmeyer H. (2001). Clock Genes in the Heart: Characterization and Attenuation with Hypertrophy. Circ. Res..

[66] Young M.E. (2023). The Cardiac Circadian Clock: Implications for Cardiovascular Disease and Its Treatment. JACC Basic Transl. Sci..

[67] van Woerden G., van Veldhuisen D.J., Westenbrink B.D., de Boer R.A., Rienstra M., Gorter T.M. (2022). Connecting Epicardial Adipose Tissue and Heart Failure with Preserved Ejection Fraction: Mechanisms, Management and Modern Perspectives. Eur. J. Heart Fail..

[68] Jeyaraj D., Haldar S.M., Wan X., McCauley M.D., Ripperger J.A., Hu K., Lu Y., Eapen B.L., Sharma N., Ficker E. (2012). Circadian Rhythms Govern Cardiac Repolarization and Arrhythmogenesis. Nature.

[69] Nuszkiewicz J., Rzepka W., Markiel J., Porzych M., Woźniak A., Szewczyk-Golec K. (2025). Circadian Rhythm Disruptions and Cardiovascular Disease Risk: The Special Role of Melatonin. Curr. Issues Mol. Biol..

[70] Grassi G. (2006). Sympathetic Overdrive and Cardiovascular Risk in the Metabolic Syndrome. Hypertens. Res..

[71] Ravussin E., Beyl R.A., Poggiogalle E., Hsia D.S., Peterson C.M. (2019). Early Time-Restricted Feeding Reduces Appetite and Increases Fat Oxidation but Does Not Affect Energy Expenditure in Humans. Obesity.

[72] Delisle B.P., Prabhat A., Burgess D.E., Ono M., Esser K.A., Schroder E.A. (2024). Circadian Regulation of Cardiac Arrhythmias and Electrophysiology. Circ. Res..

[73] He M., Wang J., Liang Q., Li M., Guo H., Wang Y., Deji C., Sui J., Wang Y.W., Liu Y. (2022). Time-Restricted Eating with or without a Low-Carbohydrate Diet Reduces Visceral Fat and Improves Metabolic Syndrome: A Randomized Controlled Trial. Cell Rep. Med..

[74] Clavero-Jimeno A., Dote-Montero M., Migueles J.H., Camacho-Cardenosa A., Medrano M., Alfaro-Magallanes V.M., Osés M., Carneiro-Barrera A., de Cabo R., Muñoz-Torres M. (2025). Time-Restricted Eating and Sleep, Mood, and Quality of Life in Adults with Overweight or Obesity: A Secondary Analysis of a Randomized Clinical Trial. JAMA Netw. Open.

[75] Cresnovar T., Habe B., Mohorko N., Kenig S., Jenko Praznikar Z., Petelin A. (2025). Early Time-Restricted Eating with Energy Restriction Improves Body Fat Mass and Cardiometabolic Risk Factors Compared with Late Time-Restricted Eating: A 3-month Randomized Clinical Trial. Clin. Nutr..

[76] Chen S., Zhang X., Kortas J., Liu H. (2025). Effects of Time-Restricted Eating on Body Composition and Metabolic Parameters in Overweight and Obese Women: A Systematic Review and Meta-Analysis. Front. Nutr..

[77] Alfaris N., Wadden T.A., Chao A.M., Alamuddin N., Berkowitz R.I. (2024). GLP-1 single, dual, and triple receptor agonists for treating type 2 diabetes and obesity: A narrative review. eClinicalMedicine.

[78] van Hout M., Forte P., Jensen T.B., Boschini C., Bækdal T.A. (2023). Effect of Various Dosing Schedules on the Pharmacokinetics of Oral Semaglutide: A Randomised Trial in Healthy Subjects. Clin. Pharmacokinet..

[79] Colonnello E., Graziani A., Rossetti R., Voltan G., Masi D., Lubrano C., Mariani S., Watanabe M., Isidori A.M., Ferlin A. (2025). The Chronobiology of Hormone Administration: “Doctor, What Time Should I Take My Medication?”. Endocr. Rev..

[80] Möller-Levet C.S., Archer S.N., Dijk D.J. (2025). Performance of Blood-Based Biomarkers for Human Circadian Pacemaker Phase: Training Sets Matter as Much as Feature-Selection Methods. J. Biol. Rhythm..

[81] Kelters I.R., Koop Y., Young M.E., Daiber A., van Laake L.W. (2025). Circadian Rhythms in Cardiovascular Disease. Eur. Heart J..

[82] Morris C.J., Purvis T.E., Hu K., Scheer F.A.J.L. (2016). Circadian Misalignment Increases Cardiovascular Disease Risk Factors in Humans. Proc. Natl. Acad. Sci. USA.

